# Diet-derived circulating antioxidants and risk of knee osteoarthritis, hip osteoarthritis and rheumatoid arthritis: a two-sample Mendelian randomization study

**DOI:** 10.3389/fmed.2023.1147365

**Published:** 2023-06-21

**Authors:** Li Huang, Yanqing Xie, Ting Jin, Mengqiao Wang, Zhen Zeng, Lina Zhang, Wenming He, Yifeng Mai, Jianmeng Lu, Han Cen

**Affiliations:** ^1^Department of Emergency Intensive Care Unit, The First Affiliated Hospital of Ningbo University, Ningbo, Zhejiang, China; ^2^Department of Cardiology, The First Affiliated Hospital of Ningbo University, Ningbo, Zhejiang, China; ^3^School of Public Health, Health Science Center, Ningbo University, Ningbo, Zhejiang, China; ^4^Institute of Geriatrics, The First Affiliated Hospital of Ningbo University, Ningbo, Zhejiang, China; ^5^Department of Second Spinal Surgery, The First Affiliated Hospital of Ningbo University, Ningbo, Zhejiang, China; ^6^Clinical Research Centre, Zhujiang Hospital, Southern Medical University, Guangzhou, Guangdong, China

**Keywords:** osteoarthritis, rheumatoid arthritis, oxidative stress, diet-derived antioxidants, Mendelian randomization

## Abstract

**Objective:**

To examine the causal associations of diet-derived circulating antioxidants with knee osteoarthritis (OA), hip OA, and rheumatoid arthritis (RA) within the two-sample Mendelian randomization (MR) framework.

**Method:**

Independent single-nucleotide polymorphisms (SNPs) significantly associated with circulating levels of diet-derived antioxidants (retinol, β-carotene, lycopene, vitamin C and vitamin E) were extracted as genetic instruments. Summary statistics of genetic instruments associated with knee OA, hip OA, and RA were obtained from corresponding genome-wide association studies (GWASs). The inverse-variance weighted (IVW) was applied as the primary analysis method, with four sensitivity analysis approaches employed to evaluate the robustness of the primary results.

**Results:**

Genetically determined per unit increment of absolute circulating levels of retinol was significantly associated with a reduced risk of hip OA [odds ratio (OR) = 0.45, 95% confidence interval (CI) 0.26–0.78, *p* = 4.43 × 10^−3^], while genetically determined per unit increase in absolute circulating levels of β-carotene was suggestively associated with increased risk of RA (OR = 1.32, 95% CI 1.07–1.62, *p* = 9.10 × 10^−3^). No other causal association was found. Significant evidence for heterogeneity and pleiotropic outlier was only identified when absolute circulating vitamin C was evaluated as the exposure, whereas all sensitive analysis provided consistently non-significant results.

**Conclusion:**

Our results demonstrated that genetically determined lifelong higher exposure to absolute circulating levels of retinol is associated with a decreased risk of hip OA. Further MR study with more genetic instruments for absolute circulating levels of antioxidants are needed to confirm our results.

## Introduction

As the two most common forms of arthritis, osteoarthritis (OA) and rheumatoid arthritis (RA) affect over 300 million and nearly 20 million people worldwide, respectively. It is foreseeable that these figures will dramatically increase as a result of the prolonged life expectancy as well as the ageing of the global population (1). OA manifests as an abnormal process of joint tissue remodeling and is now widely viewed as a disease affecting the whole joint (2). Although almost all joints could be affected, OA of the knee and hip, two commonly involved joints, contribute the most to the disease burden mainly due to joint replacement (3). RA is a chronic inflammatory autoimmune disease characterized by chronic inflammation of the synovial membrane, leading to articular cartilage and bone destruction (4). OA of the knee and hip along with RA are significantly associated with joint function loss, disability, reduced quality of life, and substantial healthcare expenditure, thereby constituting major public health challenges (3, 4). In addition, although an increasing number of anti-rheumatic disease-modifying drugs (DMARDs) are provided for RA, there is no curative drugs available presently (4), while the management of knee and hip OA is limited to symptom alleviation and total joint replacement for end stage patients (3). Therefore, in order to inform public health strategies and measures to reduce disease burden in aging population, there is an urgent need to identify modifiable risk factors and develop preventive measures against the development of knee OA, hip OA and RA.

Of note, a growing body of evidence indicates that oxidative stress associated with the abundant production of reactive oxygen species (ROS) is involved in the onset and progression of OA and RA, suggesting that diet-derived antioxidants might possess the preventive potential against OA (5) and RA (6). Oxidative stress refers to an imbalance between the production of ROS and clearance by the antioxidant defense system, leading to macromolecular damage and disruption of redox signaling and control (7). Although the exact mechanisms by which oxidative stress contributes to the initiation of OA and RA have not been completely demonstrated, accumulating evidence suggests that ROS could regulate the intracellular signaling pathways, inflammatory response, cellular apoptosis and extracellular matrix turnover (5, 6). As a consequence, it is reasonable to generate the hypothesis that adequate dietary intake of antioxidants, the scavengers of free radicals, might provide a feasible approach to prevent OA and RA. Indeed, multiple observational epidemiological studies have demonstrated that insufficient dietary intake and lower circulating levels of several common and easily accessible diet-derived antioxidants, such as retinol, β-carotene, lycopene, vitamin C and vitamin E, are significantly associated with an increased risk of OA (8–14) and RA (15–22). However, non-significant and even harmful effects of higher exposure to aforementioned diet-derived antioxidants on OA (23–28) and RA (29–32) were also reported. These conflicting results might arise from bias caused by residual confounding, reverse causality, and measurement errors commonly present in observational studies, therefore the causal associations of diet-derived antioxidants with OA and RA remains largely unclear.

Recently, Mendelian randomization (MR), employing genetic variants such as single nucleotide polymorphisms (SNPs) as instrumental variables (IVs) in observational settings to make causal inference between environmentally modifiable exposure and outcome, has been gaining popularity in recent years (33). Since genetic variants are randomly allocated during gamete formation and are independent of environmental and lifestyle factors, the estimates from MR are less susceptible to confounding bias. In addition, an individual’s germline genotype must precede the outcome of interest and genetic variants are measured with a high degree of precision, so the results derived from MR analysis are also less prone to bias resulting from reserve causality and measurement error. Therefore, we determined to evaluate the causal relationships between genetically determined lifelong diet-derived circulating antioxidants, measured as absolute circulating antioxidants as well as circulating antioxidants metabolites, and risk of knee OA, hip OA and RA within the two-sample MR framework.

## Methods

### Study design

**Figure 1:**
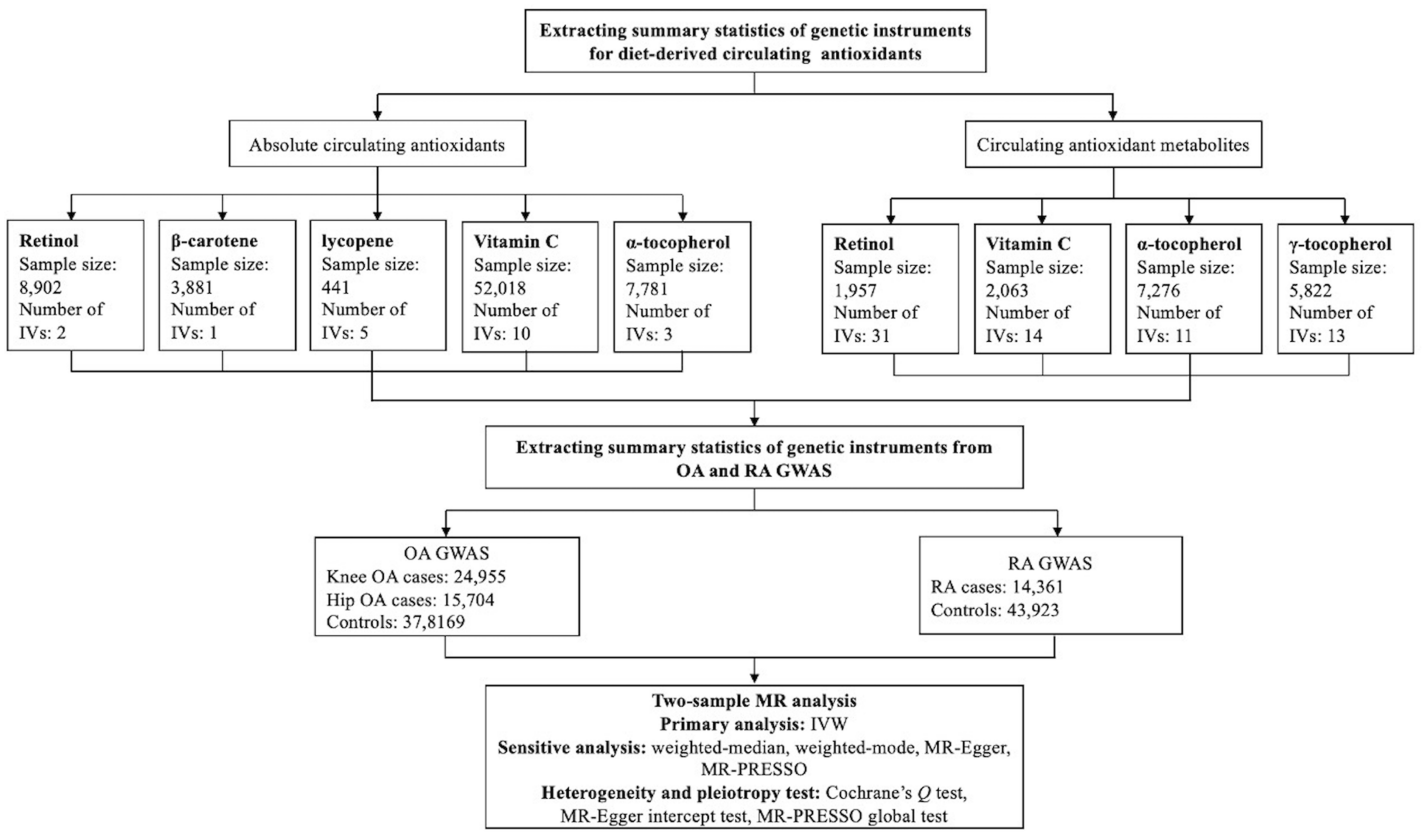
Schematic overview of our present two-sample Mendelian randomization study design. IV: instrumental variable; IVW: inverse-variance weighted; MR: Mendelian randomization; MR-PRESSO: MR pleiotropy residual sum and outlier; OA: osteoarthritis; RA: rheumatoid arthritis.

The present two-sample MR study was conducted based on publicly available genome-wide association studies (GWASs) summary-level data, and ethical approval and informed consent had been acquired in each original study. In brief, we first extracted the summary statistics of genetic instruments for absolute circulating antioxidants and circulating antioxidant metabolites from the corresponding GWASs, then the summary statistics for these genetic instruments were obtained from GWASs of knee OA, hip OA and RA. Eventually, the causal associations of diet-derived circulating antioxidants and risk of knee OA, hip OA, and RA were evaluated within a two-sample MR framework, and the schematic overview of our present study design is shown in Figure 1.

### Data sources for exposure and selection of IVs

In our present two-sample MR study, a total of five common diet-derived antioxidants, namely retinol, β-carotene, lycopene, vitamin C and Vitamin E (α-tocopherol and γ-tocopherol), were included. For each diet-derived antioxidant, the absolute circulating level and/or relative circulating level of the antioxidant metabolite were considered. Summary-level genetic association statistics for independent SNPs (*r*^2^ < 0.001 within a 10,000 kb sliding window) serving as IVs significantly associated with diet-derived circulating antioxidants were extracted.

As for absolute circulating antioxidants, we obtained appropriate SNPs applied as genetic instruments for retinol, β-carotene, lycopene, vitamin C, and α-tocopherol. For retinol, two independent SNPs significantly associated with serum retinol levels (*p* < 5 × 10^−8^) in the meta-analysis of a GWAS and further replication studies comprising 8,902 Caucasian individuals were selected (34). For β-carotene, one SNP significantly associated with plasma levels of β-carotene (*p* < 5 × 10^−8^) in meta-analysis of GWAS and further replication samples involving 3,881 European individuals was selected (35). For lycopene, a total of five independent SNPs significantly linked to serum lycopene concentrations at sub genome-wide significance (*p* < 5 × 10^−6^) were obtained from a GWAS of 441 Old Order Amish adults (36). For vitamin C, a total of 10 independent SNPs found to be significantly associated with plasma vitamin C levels (*p* < 5 × 10^−8^) in a large-scale GWAS in 52,018 individuals of European ancestry were chosen (37). For α-tocopherol, three independent SNPs shown to be significantly associated with circulating alpha-tocopherol concentrations (*p* < 5 × 10^−8^) in meta-analysis of one GWAS with two independent replication samples consisting of 7,781 European individuals were obtained (38).

Regarding circulating antioxidant metabolites, appropriate SNPs employed as genetic instruments for retinol, vitamin C, α-tocopherol and γ-tocopherol were obtained. Independent SNPs associated with blood metabolites of vitamin C (*n* = 14), α-tocopherol (*n* = 11), and γ-tocopherol (*n* = 13) at suggestive genome-wide significance level (*p* < 1 × 10^−5^) were derived from a GWAS comprising 7,824 adults from 2 European population studies (39). A total of 31 independent SNPs associated with blood metabolite of retinol at suggestive genome-wide significance level (*p* < 1 × 10^−5^) were derived from a recently published GWAS comprising 1,957 European participants (40).

All selected genetic instruments mentioned above have been used in recent MR studies, and all have *F*-statistic greater than 10, indicating that these genetic instruments are not weak instruments (41–43).

### Data sources for outcomes

Summary statistics for the associations of genetic instruments with knee OA (*n* = 24,955) and hip OA (*n* = 15,704) were extracted from one recent GWAS meta-analysis performed based on the United Kingdom Biobank and Arthritis Research UK Osteoarthritis Genetics (arcOGEN) resources, in which approximately 17.5 million SNPs in 455,221 individuals of European ancestry were analyzed (44). In United Kingdom Biobank, OA cases were hospital diagnosed, and the cases in arcOGEN were defined according to radiographic finding and/or total joint replacement (45). Summary statistics for the associations of genetic instruments with RA were derived from a GWAS meta-analysis comprising 14,361 RA cases and 43,923 controls of the European ancestry (45). All patients with RA either satisfied the 1987 American College of Rheumatology (ACR) revised criteria for the classification of RA (46) or were diagnosed by board-certified rheumatologists.

### Statistical analysis

The inverse-variance weighted (IVW) was employed as the primary MR analysis approach, and four sensitive analysis methods, including weighed median, weighted mode, MR-Egger and MR pleiotropy residual sum and outlier (MR-PRESSO), were applied to test the robustness of IVW estimates to horizontal pleiotropy. When all selected SNPs are valid instrumental variables, the IVW method gives the most precise estimate of the causal effect by combing the Wald ratio estimate of each genetic instrument in a meta-analysis model. When only one genetic instrument is available, the Wald ratio estimate was applied. The weighted median method could provide a consistent causal estimate if at least one half of the employed genetic instruments are valid (47), while the weighted mode approach could produce consistent estimate when the majority of similar individual-instrument effect estimates derives from valid instruments (48). MR-Egger regression can detect horizontal pleiotropy by its intercept test as well as provide pleiotropy-corrected estimate (49). MR-PRESSO test can detect potential outlying SNPs and provide causal estimate after removal of outliers (50). Cochrane’s *Q* statistic was applied to evaluate the heterogeneity among Wald ratio estimates from genetic instruments. The minimum detectable odds ratios (ORs) based on a type I error being 0.05, a statistical power of 0.8, sample size, proportion of case and variance explained by genetic instruments (*R^2^*) were estimated using an online tool^1^ (51). The values of *R*^2^ were either extracted from the original studies or calculated based on the following formula: *R*^2^ = 2 × EAF × (1 − EAF) × beta^2^/ [(2 × EAF × (1 − EAF) × beta^2^ + 2 × EAF × (1 − EAF) × *N* × se^2^)], where EAF, beta, se, and *N* denote the effect allele frequency, estimated effect and its standard error of genetic instrument on exposure, and sample size, respectively (52). The effect estimates of per unit (specifically, μg/L in natural log-transformed scale for retinol, μmol/L in natural log-transformed scale for β-carotene, μg/dL for lycopene, μmol/L in standard deviation [SD] for vitamin C, mg/L in log-transformed scale for α-tocopherol) increment in absolute circulating antioxidants or per 10-fold increase in circulating antioxidant metabolites on knee OA, hip OA and RA were expressed as expressed as ORs with 95% confidence intervals (CIs). A two-tailed *p* value less than 5.56 × 10^−3^ (0.05/9), set based on Bonferroni correction method, was considered statistically significant, while a two-sided *p* value less than 0.05 was regarded suggestive significance. All analysis were performed utilizing “forestplot,” “TwoSampleMR” and “MRPRESSO” packages in R version 4.2.0.

## Results

Table 1 presents the summary information of data sources for the selection of genetic instruments used in our present study, and the variance collectively explained by genetic instruments (*R^2^*) for absolute circulating levels of retinol, β-carotene, lycopene, vitamin C, and α-tocopherol was 2.3, 2.5, 31.0, 1.7, and 1.7%, respectively. The *R*^2^ for circulating metabolites levels of retinol, vitamin C, α-tocopherol, and γ-tocopherol was 22.9, 21.7, 6.8, and 9.8%, respectively. The effect estimates for the associations of genetic instruments for absolute circulating antioxidants and circulating antioxidant metabolites with knee OA, hip OA and RA are shown in Supplementary Tables 2, 3, respectively. Given a type I error of 5% and a statistical power of 0.80, the minimum detectable ORs are shown in Table 2.

**Table 1:** Summary of data sources for the selection of genetic instruments as proxies for diet-derived circulating antioxidants in our present two-sample Mendelian randomization (MR) study.

Phenotype	Study type	Sample size	No. of SNPs	LD (*r^2^*)	*P*-value	Unit	Ancestry	Explained variance (*R^2^*)^†^	PMID
Absolute circulating antioxidants									
Retinol	GWAS and replication	8,902	2	0.001	5 × 10^-8^	μg/L in natural log-transformed scale	European	2.3%^**‡**^	21878437
β-carotene	GWAS	3,881	1	NA	5 × 10^-8^	μmol/L in natural log-transformed scale	European	2.5%	19185284
Lycopene	GWAS	441	5	0.001	5 × 10^-6^	μg/dL	European	31.0%	26861389
Vitamin C	GWAS	52,018	10	0.001	5 × 10^-8^	μmol/L in standard deviation (SD) scale	European	1.7%	33203707
α-tocopherol	GWAS and replication	7,781	3	0.001	5 × 10^-8^	mg/L in log-transformed scale	European	1.7%^‡^	21729881
Circulating antioxidant metabolites									
Retinol	GWAS	1,957	31	0.001	1 × 10^-5^	log10-transformed metabolites	European	22.9%	28263315
Vitamin C	GWAS	2,063	14	0.001	1 × 10^-5^	log10-transformed metabolites	European	21.7%	24816252
α-tocopherol	GWAS	7,276	11	0.001	1 × 10^-5^	log10-transformed metabolites	European	6.8%	24816252
γ-tocopherol	GWAS	5,822	13	0.001	1 × 10^-5^	log10-transformed metabolites	European	9.8%	24816252

LD: linkage disequilibrium; NA: not applicable.

† Explained variance (*R^2^*) was either derived from the original study or calculated based on the following formula: *R*^2^ = 2 × EAF × (1−EAF) × *beta*^2^ /[(2 × EAF × (1−EAF) × *beta*^2^ + 2 × EAF × (1 − EAF) × *N* × *se*^2^)], where EAF, beta, se, and N denote the effect allele frequency, estimated effect and its standard error of genetic instrument on exposure, and sample size, respectively.

‡ These values of *R*^2^ were extracted from original studies, and the others were calculated based on the formula mentioned above.

**Table 2:** Minimum detectable odds ratios (ORs) for associations of genetically determined per unit increase in diet-derived circulating antioxidants with risk of knee osteoarthritis (OA), hip OA and rheumatoid arthritis (RA).

Outcome	Sample size	Proportion of cases	Absolute circulating antioxidants					Circulating antioxidant metabolites			
			Retinol	β-carotene	Lycopene	Vitamin C	α-tocopherol	Retinol	Vitamin C	α-tocopherol	γ-tocopherol
Knee OA	403,124	6.19%	0.88/1.12	0.89/1.12	0.97/1.03	0.86/1.14	0.86/1.14	0.96/1.04	0.96/1.04	0.93/1.07	0.94/1.06
Hip OA	393,873	3.99%	0.85/1.15	0.86/1.15	0.96/1.04	0.83/1.17	0.83/1.17	0.95/1.05	0.95/1.05	0.91/1.09	0.93/1.07
RA	58,284	24.64%	0.83/1.18	0.84/1.18	0.95/1.05	0.81/1.22	0.81/1.22	0.94/1.06	0.94/1.06	0.90/1.11	0.92/1.09

Assuming a type I error being 5% and a statistical power of 0.80, the minimal ORs were calculated based on an online tool: http://cnsgenomics.com/shiny/mRnd/. The variance totally explained by genetic instruments for absolute circulating levels of retinol, β-carotene, lycopene, vitamin C and α-tocopherol was 2.3%, 2.5%, 31.0%, 1.7%, and 1.7%, respectively. The variance collectively explained by genetic instruments for circulating metabolites of retinol, vitamin C, α-tocopherol and γ-tocopherol was 22.9%, 21.7%, 6.8%, and 9.8%, respectively. The ORs represent the effect estimates of per unit (specifically, μg/L in natural log-transformed scale for retinol, μmol/L in natural log-transformed scale for β-carotene, μg/dL for lycopene, μmol/L in standard deviation [SD] for vitamin C, mg/L in log-transformed scale for α-tocopherol) increment in absolute circulating antioxidants or per 10-fold increase in circulating antioxidant metabolites on knee OA, hip OA and RA.

### Causal associations of absolute circulating antioxidants with OA and RA

**Figure 2:**
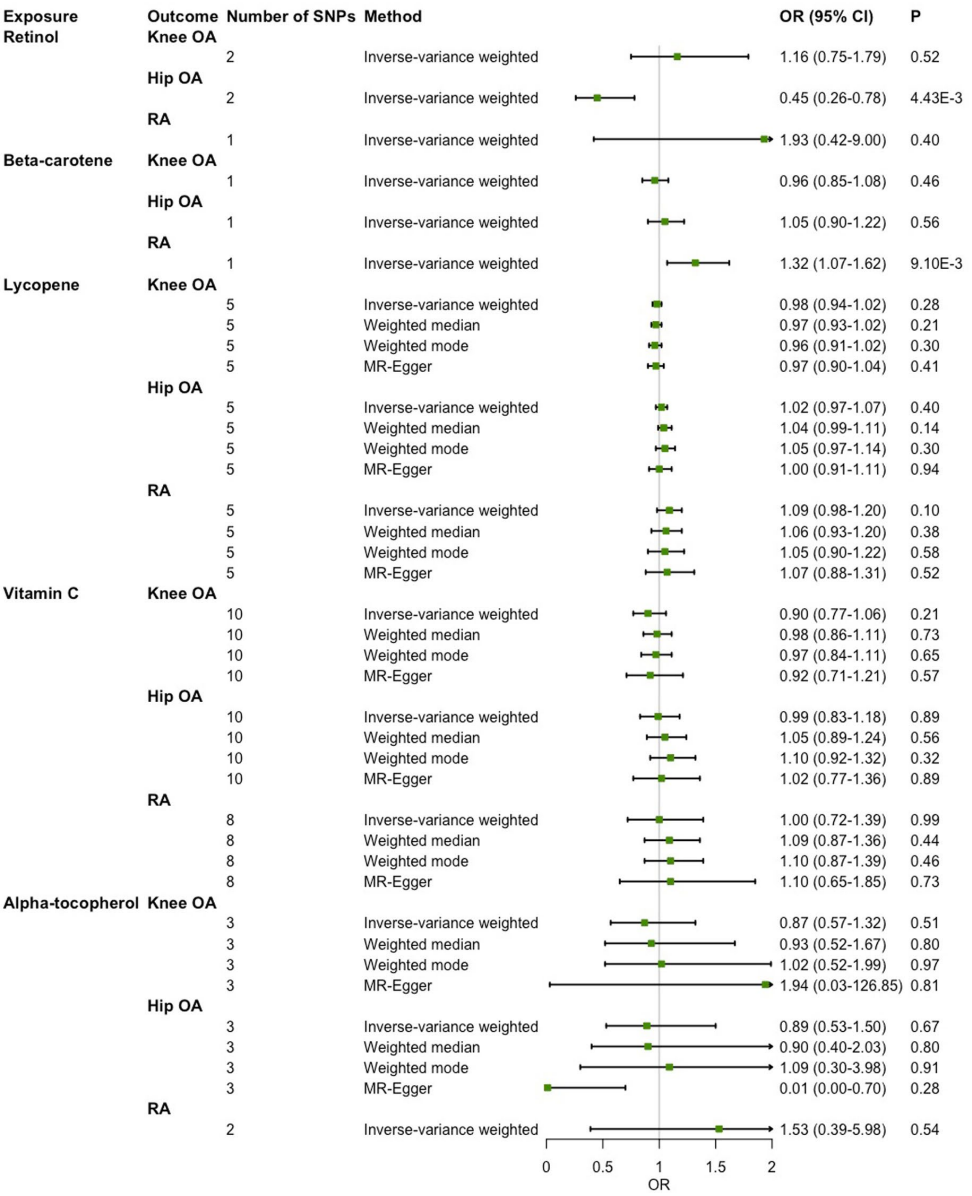
Two-sample Mendelian randomization (MR) analysis results of the causal relationships between genetically determined absolute circulating antioxidants and risk of knee osteoarthritis (OA), hip OA and rheumatoid arthritis (RA).

As shown in Figure 2, in the main IVW analysis, genetically determined per unit increment of retinol was significantly associated with reduced risk of hip OA (OR = 0.45, 95% CI 0.26–0.78, *p* = 4.43 × 10^−3^), while genetically determined per unit increase in β-carotene was associated with increased risk of RA (OR = 1.32, 95% CI 1.07–1.62, *p* = 9.10 × 10^−3^) with suggestive significance. Since the number of genetic instruments available for retinol and β-carotene was less than 3, sensitive analysis could not be performed. As for the associations of the other three diet-derived antioxidants (lycopene, vitamin C and α-tocopherol) with knee OA, hip OA and RA, no significant evidence was found in the main IVW analysis, and the estimates derived from all sensitive analysis methods were consistently non-significant. Significant heterogeneity across individual genetic instrument estimate was found for the association of vitamin C with knee OA (*p* = 2.09 × 10^−3^), hip OA (*p* = 0.03) and RA (*p* = 1.91 × 10^−3^). No significant evidence of pleiotropy was found in MR-Egger regression (all *p* for MR-Egger intercept test > 0.05; Table 3). While outlier was identified in the MR-PRESSO analysis for the associations of genetically determined per unit increase in vitamin C with knee OA and RA, the outlier-corrected estimate did not change substantially (Supplementary Table 4).

**Table 3:** The results of heterogeneity and pleiotropy test for two-sample Mendelian randomization (MR) analysis of the causal relationships between genetically determined absolute circulating antioxidants and risk of knee osteoarthritis (OA), hip OA and rheumatoid arthritis (RA).

Exposure	Outcome	IVW		MR-Egger	MR-PRESSO
		Cochrane’s Q statistic	*P*-value	Intercept test *P*-value	Global test *P*-value
Retinol	Knee OA	0.09	0.76	NA	NA
	Hip OA	0.30	0.58	NA	NA
	RA	NA	NA	NA	NA
β-carotene	Knee OA	NA	NA	NA	NA
	Hip OA	NA	NA	NA	NA
	RA	NA	NA	NA	NA
Lycopene	Knee OA	1.98	0.74	0.69	0.70
	Hip OA	3.81	0.43	0.73	0.50
	RA	1.25	0.87	0.90	0.86
Vitamin C	Knee OA	25.94	2.09 × 10^-3^	0.84	0.02
	Hip OA	19.01	0.03	0.78	0.07
	RA	22.82	1.83 × 10^-3^	0.63	0.03
α-tocopherol	Knee OA	2.06	0.36	0.77	NA
	Hip OA	5.74	0.06	0.28	NA
	RA	1.67	0.20	NA	NA

NA: not applicable; OA: osteoarthritis; RA: rheumatoid arthritis.

### Causal associations of circulating antioxidant metabolites with OA and RA

**Figure 3:**
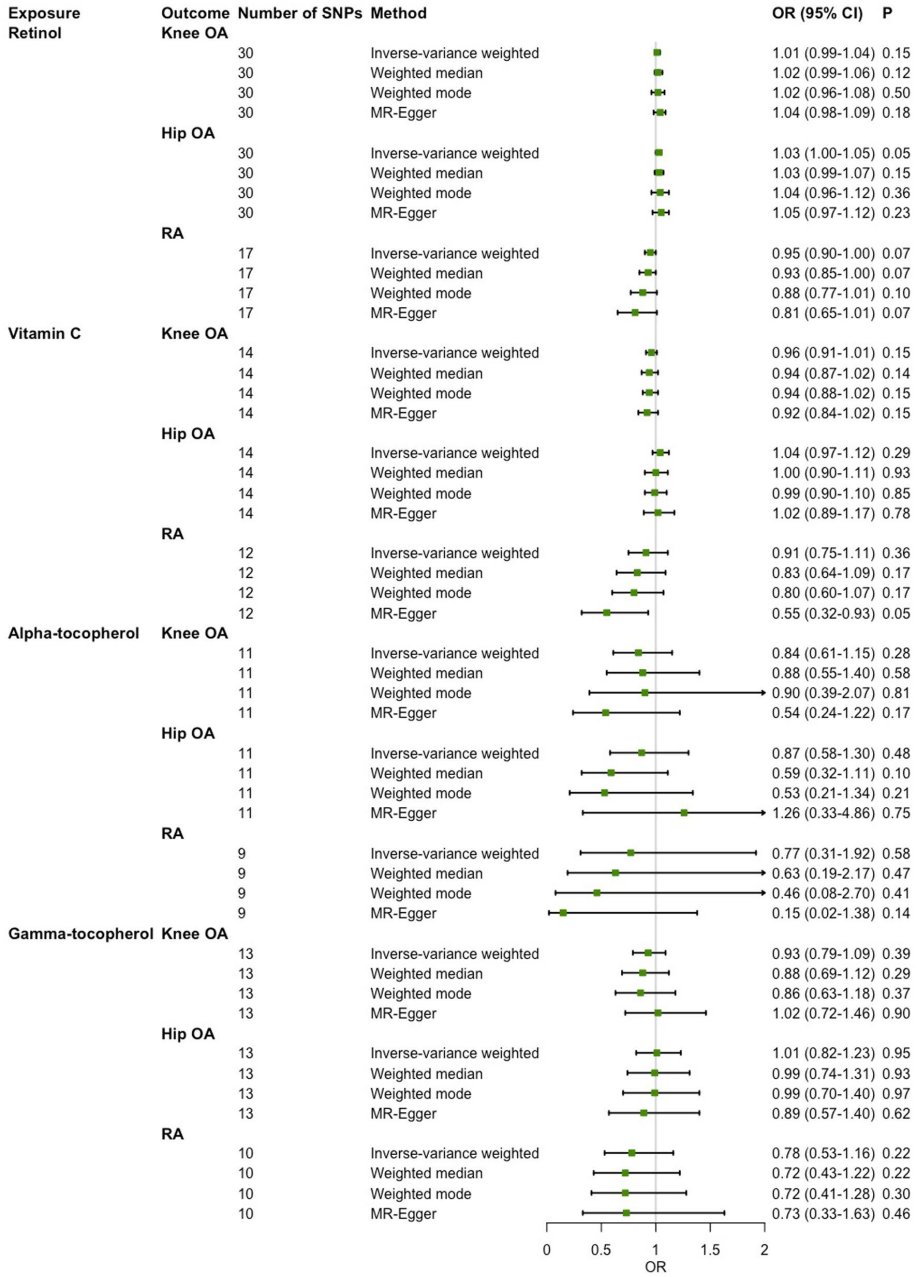
Two-sample Mendelian randomization (MR) analysis results of the causal relationships between genetically determined circulating antioxidant metabolites and risk of knee osteoarthritis (OA), hip OA and rheumatoid arthritis (RA).

In the main IVW analysis, no significant evidence was detected for associations of genetically determined circulating antioxidant metabolites with risk of knee OA, hip OA and RA (Figure 3). The estimates derived from all sensitive analysis were consistent with the findings of main IVW analysis, with all associations being non-significant. No evidence of heterogeneity across genetic instrument estimates was found. Moreover, according to the MR-Egger intercept test and MR-PRESSO global test, no significant evidence of horizontal pleiotropy was found (Table 4).

**Table 4:** The results of heterogeneity and pleiotropy test for two-sample Mendelian randomization (MR) analysis of the causal relationships between genetically determined circulating antioxidant metabolites and risk of knee osteoarthritis (OA), hip OA and rheumatoid arthritis (RA).

Exposure	Outcome	IVW		MR-Egger	MR-PRESSO
		Cochrane’s Q statistic	*P*-value	Intercept test *P*-value	Global test *P*-value
Retinol	Knee OA	30.50	0.39	0.40	0.39
	Hip OA	37.02	0.15	0.59	0.14
	RA	22.89	0.12	0.15	0.14
Vitamin C	Knee OA	8.61	0.80	0.40	0.81
	Hip OA	14.29	0.35	0.75	0.37
	RA	14.34	0.21	0.07	0.26
α-tocopherol	Knee OA	12.13	0.28	0.27	0.25
	Hip OA	18.60	0.05	0.57	0.05
	RA	6.75	0.56	0.16	0.45
γ-tocopherol	Knee OA	9.08	0.70	0.57	0.64
	Hip OA	10.46	0.58	0.56	0.62
	RA	8.47	0.49	0.84	0.42

NA: not applicable; OA: osteoarthritis; RA: rheumatoid arthritis.

## Discussion

Utilizing genetic variants strongly and significantly associated with the levels of diet-derived circulating antioxidants assessed as absolute levels and relative concentrations as instrumental variables, our present two-sample MR analysis suggests that genetically determined lifelong higher exposure to absolute circulating levels of retinol is associated with a reduced risk of hip OA, while genetically predicted lifetime increased absolute circulating levels of β-carotene is suggestively associated with higher risk of RA.

Osteoarthritis of the knee and hip usually leads to joint replacement and constitute one leading cause of disability, thereby contributing the most to the disease burden of OA (4). Since no curative and disease-modifying drugs are available, with the aging of the population and increased prevalence of obesity, it is of great public health significance to seek for potential preventive measures against knee and hip OA. During the past decades, dietary modification explored as potential preventive measure for OA has attracted increasing attention owing to its good feasibility (53). For instance, the results derived from cross-sectional analysis carried out by Hirst-Jones et al. based on TWINS-UK cohort revealed that ‘Traditional English’ dietary pattern was associated with increased risk of hand OA while Mediterranean dietary pattern found to be protective against OA (54). Of note, in view of the significant role of oxidative stress accompanied by the excessive generation of ROS in the pathogenesis of OA (5), a series of observational studies have been conducted to explore the associations of diet-derived antioxidants, assessed in the form of dietary intake or circulating concentrations, with the risk of OA, and controversial results have been reported (8–14, 23–28). As for retinol, based on the data of Comprehensive Health Examination Program (CHEP, Yakumo Study) conducted in rural Japanese inhabitants, one cross-sectional study did not find a significant association between serum retinol levels, quantified as tertiles, and radiographic knee OA (13), while one recent cohort study demonstrated that the baseline serum levels of retinol treated as tertiles was only positively and significantly associated with incidence of knee OA among females but not in the whole cohort over the ensuing 10 years (8). Consistently, we did not find significant evidence for a causal relationship between genetically determined circulating levels of retinol and knee OA. Of note, as the same as one recent MR analysis based on the same GWASs data sources for absolute circulating retinol levels and OA, genetically determined higher absolute circulating levels of retinol was associated with a reduced risk of hip OA (55), whereas non-significant evidence was detected for circulating metabolites of retinol. As for β-carotene, the results of our MR analysis were consistent with most studies showing non-significant evidence for an association of dietary intake of β-carotene with OA (23) and knee OA (11, 28) as well as serum levels of β-carotene with prevalence (13) and incidence of knee OA (8), although one matched case–control study showed that those in the highest tertile of trans-beta-carotene were more likely to have knee OA (27). As for lycopene, no significant evidence for an association of serum lycopene levels with knee OA was detected neither in the cross-sectional study (13) nor in the cohort study (8) carried out based on the data of CHEP among Japanese. Moreover, a matched case–control study did not find a significant difference in serum concentrations of lycopene between 200 patients with radiographic knee OA and 200 controls (27), while one small sample size cross-sectional case–control study consisting of 35 primary knee OA patients and 35 matched healthy controls found that serum concentrations of lycopene were significantly lower in knee OA patients in comparison with controls (9). In line with the non-significant evidence detected in most studies, we did not find significant causal effect of absolute circulating lycopene levels on knee OA and hip OA. As for vitamin C and vitamin E, although protective roles of higher exposure have been reported in several studies (9–14), non-significant and even harmful effects were found in several other studies (8, 23–26, 28). In agreement with one recent meta-analysis suggesting that the intake of vitamin C and vitamin E was not significantly associated with knee OA (56), we did not find significant evidence for causal effects of absolute circulating levels and circulating metabolites of vitamin C, and vitamin E on risk of knee OA as well as hip OA. Collectively, the robust non-significant causal associations of genetically determined circulating levels of β-carotene, lycopene, vitamin C and vitamin E with the risk of knee OA and hip OA suggest that variations in lifetime exposure to these antioxidants have no effect on the development of these diseases.

Similar to OA, the potential protective roles of diet-derived antioxidants against the development of RA have also attracted extensive attention during the past decades. Many observational studies have been carried out to examine the relationships between these diet-derived antioxidants in terms of dietary intake or circulating levels and RA with controversial results (15–22, 29–32), and no conclusive statements can be made. As for retinol, results regarding the association of its circulating level with RA produced consistently non-significant evidence (18, 22), while studies on the association of its dietary intake with RA showed mixed results (19, 29, 31). Specifically, intake of retinol was not found to be significantly associated with the risk of developing RA in cohort studies (31), while one cross-sectional study found that insufficient intake of retinol was associated with a lower risk of RA (29) and another cross-sectional case–control study demonstrated that energy-adjusted intake of retinol was significantly lower in RA patients than controls (19). As for β-carotene, a nested case–control study found that incident cases of rheumatoid arthritis had lower pre-diagnostic serum levels of β-carotene than matched controls (22), and another matched case–control study demonstrated that the plasma concentrations of β-carotene was significantly lower in RA patients compared with healthy controls (16). Whereas another nested case–control study (30) and one cross-sectional study conducted in America did not find significant association of plasma levels of β-carotene with RA (18). Besides, studies on the association of β-carotene intake with RA also provided conflicting results, with non-significant results detected in two cohort studies (20, 31) and a significant signal shown in one cross-sectional case–control study (19). With respect to lycopene, results regarding association of its intake with RA were consistently non-significant as found in two cohort studies (20, 31), whereas the association of its circulating levels with RA was significant in one cross-sectional (18) and non-significant in one nested case–control study (30). Regarding vitamin C, its circulating levels were shown to be significantly down-regulated in RA patients in one small cross-sectional case–control study comprising 20 RA patients and 20 healthy controls (15), while in another large cross-sectional study including 5,302 subjects, no significant difference in plasma concentrations of vitamin C between RA patients and non-RA participants was found (18). Intake of vitamin C was not found to be significantly associated with the risk of developing RA in two cohort studies (20, 31), while two cross-sectional studies reported conflicting results, with non-significant evidence found in a large (*n* = 8,789) study (29) and significant evidence detected in one small study (*n* = 108) (17). As regard vitamin E, studies on association of its intake with RA produced consistently non-significant results (20, 29, 31, 32), while controversial results were reported for the association of its circulating levels with RA (18, 19, 21, 22). Specifically, one cross-sectional case–control study found that RA patients had lower plasma levels of α-tocopherol than controls (19), while another cross-sectional study did not find a significant difference in plasma α-tocopherol levels between RA patients and non-RA individuals (18). Besides, one nested case–control found that individuals had highest tertile of serum α-tocopherol levels were less likely to develop RA compared with those who had lowest tertile of serum levels of α-tocopherol (21), whereas another nested case–control study did not find a significant difference in baseline serum α-tocopherol concentrations between incident RA cases and controls (22). The discrepancy among different studies on the associations of diet-derived antioxidants included in our study with the risk of RA may arise from the variation in many aspects such as study design, sample size and exposure measurement method (dietary intake and circulating levels). In addition, observational studies are subject to biases that limit causal inference, including residual confounding, reverse causation and measurement errors. Leveraging genetic instruments as proxies for diet-derived circulating antioxidants, we applied two-sample MR analysis to avoid biases frequently present in observational studies and make causal inference about the relationships between diet-derived circulating antioxidants, assess as absolute circulating antioxidants and circulating antioxidant metabolites, and risk of RA. However, we only detected suggestive evidence for association of higher levels of absolute circulating levels of β-carotene with increased risk of RA. Although the biological mechanism underlying this association remains unclear, β-carotene supplementation has also been reported to be associated with increased overall cardiovascular incidence and mortality (57) as well as increased risk of lung cancer mortality in recent meta-analysis (58). It has been shown that antioxidants function as reducing agents would experience a shift towards pro-oxidant activity, therefore the pro-oxidant activity of beta-carotene has been regarded as one possible explanation for its harmful health effect (59). Of interest, the study performed by Palozza et al. (60) found that beta-carotene might enhance smoking-induced oxidative stress and play potential deleterious roles at the pO2 normally present in lung tissue. Since smoking, to date, is the most well-characterized environmental risk factor of RA (61), it is of great interest to perform factorial MR in the future to examine whether there is synergistic effect between higher circulating levels of beta-carotene and smoking on the risk of RA. Further population-based studies are needed to confirm this suggestive association result. It is worth mentioning that one recently published MR study performed by Zhou et al. (62) also focused on the causal relationships between diet-derived antioxidants and risk of RA. Different from our present MR study, another GWAS dataset of RA derived from FinnGen consortium in addition to the RA GWAS dataset utilized by us was included in that study. Moreover, proxy SNPs were applied when SNPs employed as IVs were absent in the RA GWAS dataset (62). These might mainly account for the difference in the observed associations between genetically determined circulating levels of retinol metabolite and risk of RA. Genetically determined higher levels of circulating retinol metabolite was found to be significantly associated with reduced risk of RA in the MR study conducted by Zhou et al. (62), and our study detected a trend towards significant association results derived from all MR estimate methods except for weighted mode method.

The main strength of our present study lies in the utilization of genetic instruments as proxies for diet-derived circulating antioxidants evaluated as absolute levels and relative concentrations in the form of metabolites to perform two-sample MR analysis to infer their causal effects on risk of knee OA, hip OA and RA. However, several potential limitations should be acknowledged. First, since only less than three genetic instruments are available for absolute circulating levels of retinol and β-carotene, we could not perform sensitive analysis to examine and correct for the potential pleiotropy. Nevertheless, these genetic instruments are not associated with other factors as shown in PhenoScanner database, suggesting that the possibility of pleiotropy is small. Second, non-linear causal associations of diet-derived circulating antioxidants with OA and RA could not be examined based on the summary-level data. However, it is reasonable to speculate that insufficient or excessive levels of antioxidants might have a harmful health effect. Third, our present MR analysis was underpowered to detect small causal effects due to the small proportion of variance explained by currently available genetic instruments for absolute circulating levels of all included antioxidants except for lycopene. Notwithstanding, consistently non-significant evidence was shown for circulating metabolites with sufficient statistical power. Last, although most of the causal associations detected in our present MR analysis were non-significant, we could not rule out the possibility that diet-derived antioxidants might have beneficial health effects in individuals deficient in these antioxidants. Besides, a synergistic effect between diet-derived antioxidants might exist, whereas this possibility could not be explored in our present MR study.

Taken together, utilizing genetic instruments as proxies for diet-derived circulating antioxidants evaluated as absolute levels and relative levels of their metabolites, the causal associations of diet-derived circulating antioxidants with the risk of knee OA, hip OA and RA were comprehensively assessed within a two-sample MR framework. We found that genetically predicted higher absolute circulating levels of retinol are associated with a reduced risk of hip OA, while genetically predicted higher absolute circulating levels of β-carotene are associated with increased risk of RA. Further MR study with more genetic instruments for absolute circulating levels of antioxidants, followed by the findings in larger-scale GWASs in the future, are needed to confirm our results.

## Data availability statement

The original contributions presented in the study are included in the article/Supplementary Material, and further inquiries can be directed to the corresponding author.

## Ethics statement

Ethical approval was not provided for this study on human participants because the present two-sample MR study was conducted based on publicly available genome-wide association studies (GWASs) summary-level data, and ethical approval and informed consent had been acquired in each original study.

## Author contributions

YM, JL, and HC had full access to all of the data in the study and took responsibility for the integrity of the data and the accuracy of the data analysis. LH, YM, JL, and HC contributed to conception and design. LH, YX, TJ, ZZ, MW, and HC were involved in the acquisition of data. LH, YM, JL, and HC carried out analysis and interpretation of data. All authors were involved in drafting the article or revising it critically for important intellectual content, and approved the final version to be submitted for publication.

## Funding

This work was supported by Zhejiang Provincial Natural Science Foundation of China (Grant No. LY20H260001) and K.C. Wong Magna Fund in Ningbo University.

## Conflict of interest

The authors declare that the research was conducted in the absence of any commercial or financial relationships that could be construed as a potential conflict of interest.

## Publisher’s note

All claims expressed in this article are solely those of the authors and do not necessarily represent those of their affiliated organizations, or those of the publisher, the editors and the reviewers. Any product that may be evaluated in this article, or claim that may be made by its manufacturer, is not guaranteed or endorsed by the publisher.

## Footnotes

^1^ http://cnsgenomics.com/shiny/mRnd/
