# Supplementary material for: Diet-derived circulating antioxidants and risk of knee osteoarthritis, hip osteoarthritis and rheumatoid arthritis: a two-sample Mendelian randomization study
Source: Front Med (Lausanne). 2023 Jun 21;10:1147365. doi: 10.3389/fmed.2023.1147365 (PMC10321672; doi:10.3389/fmed.2023.1147365)
Supplement: Supplementary file 1 [file Table_1.docx]

Supplementary Material

Article Title Diet-Derived Circulating Antioxidants and Risk of Knee Osteoarthritis, Hip Osteoarthritis and Rheumatoid Arthritis: A Two-Sample Mendelian Randomization Study

**Li Huang, Yanqing Xie, Ting Jin, Mengqiao Wang, Zhen Zeng, Lina Zhang, Wenming He, Yifeng Mai^*^, Jianmeng Lu^*^, Han Cen^*^**

*** Correspondence:** Yifeng Mai: fymaiyifeng@nbu.edu.cn; Jianmeng Lu: ningguxiansheng@163.com; Han Cen: cenhan@smu.edu.cn

**Supplementary table 1** Summary of measurement method, concentration of antioxidants, age and sex of data sources for the selection of genetic instruments as proxies for diet-derived circulating antioxidants in our present two-sample Mendelian randomization (MR) study

| **Phenotype** | **Sample size** | **Measurement method** | **Concentration^†^** | **Age (years)^††^** | **Sex (male, %)** | **PMID** |
| --- | --- | --- | --- | --- | --- | --- |
| Absolute circulating antioxidants | | | | | | |
| Retinol | 8,902 | ATBC: reversed-phase liquid chromatography with diode-array UV detection  PLCO: reversed-phase liquid chromatography with diode-array UV detection  NHS: reversed-phase high-performance liquid chromatography with UV detection with modifications.  InCH: isocratic high-performance liquid chromatography | ATBC: 572 (496–654) ug/L  PLCO: 672 (562–794) ug/L  NHS: 563 (485–657) ug/L  InCH: 538 (461–634) ug/L | ATBC:58 (54–62)  PLCO: 65 (61–68)  NHS: 60 (55–64)  InCH: 71 (66–77) | GWAS studies:  ATBC:100  PLCO: 100  Replication studies:  NHS: 0  InCH: 45 | 21878437 |
| β-carotene | 3,881 | InCHIANTI and WHAS: high-performance liquid chromatography  ATBC: reverse-phase liquid chromatography | InCHIANTI: 0.41 (0.27) umol/L  WHAS I: 0.42 (0.38) umol/L  WHAS II: 0.57 (0.54) umol/L  ATBC: 0.40 (0.30) umol/L | NA | NA | 19185284 |
| Lycopene | 441 | reverse-phase high-pressure liquid chromatography | 39.2 (19.9) ug/dL | 43.1 (13.0) | 58 | 26861389 |
| Vitamin C | 52,018 | EPIC-InterAct and EPIC-CVD: high-performance liquid chromatography with ultraviolet detection  EPIC-Norfolk and Fenland: fluorometric assay | Fenland GWAS array: 66.2 (21.3) umol/L  Fenland UKBB array: 68.6 (21.5) umol/L  Fenland Core- Exome array: 68.3 (21.8) umol/L  InterAct subcohort GWAS array: 42.8 (19) umol/L  InterAct subcohort core-exome array: 42.9 (19.1) umol/L  InterAct non-subcohort GWAS array: 36.4 (17.6) umol/L  InterAct non-subcohort core-exome array: 36.5 (18.9) umol/L  EPIC- Norfolk GWAS array: 53.8 (20.2) umol/L  EPIC-CVD subcohort: 41.0 (21.0) umol/L  EPIC-CVD non-subcohort: 37.9 (20.8) umol/L | Fenland GWAS array: 45 (7)  Fenland UKBB array: 49 (7)  Fenland Core- Exome array: 51 (7)  InterAct subcohort GWAS array: 51 (9)  InterAct subcohort core-exome array: 53 (9)  InterAct non-subcohort GWAS array: 55 (8)  InterAct non-subcohort core- exome array: 56 (7)  EPIC- Norfolk GWAS array: 59 (9)  EPIC-CVD subcohort: 53 (12)  EPIC-CVD non-subcohort: 57 (8) | Fenland GWAS array: 44  Fenland UKBB array: 47  Fenland Core- Exome array: 45  InterAct subcohort GWAS array: 35  InterAct subcohort core-exome array: 38  InterAct non-subcohort GWAS array: 48  InterAct non-subcohort core- exome array: 52  EPIC- Norfolk GWAS array: 47  EPIC-CVD subcohort: 41  EPIC-CVD non-subcohort: 55 | 33203707 |
| α-tocopherol | 7,781 | ATBC: high-performance liquid chromatography  PLCO: CLIA  NHS: reversed-phase high-performance liquid chromatography | ATBC: 11.9 (3.4) mg/L  PLCO: 19.1 (9.7) mg/L  NHS: 13.3 (5.8) mg/L | ATBC: 58.1 (5.0)  PLCO: 64.6 (4.9)  NHS: 59.2 (6.3) | ATBC: 100  PLCO: 100  NHS: 0 | 21729881 |
| Circulating antioxidant metabolites | | | | | | |
| Retinol | 1,957 | The non-targeted metabolomics analysis was performed at Metabolon (Durham, North Carolina, USA) on a platform consisting of four independent ultra-high-performance liquid chromatography–tandem mass spectrometry instruments | NA | 58 (32–87) | NA | 28263315 |
| Vitamin C | 2,063 | liquid­phase chromatography and gas chromatography separation coupled with tandem mass spectrometry | NA | TwinsUK: 53.4 (14.0)  KORA F4: 60.8 (8.8) | TwinsUK: 7  KORA F4: 49 | 24816252 |
| α-tocopherol | 7,276 | liquid­phase chromatography and gas chromatography separation coupled with tandem mass spectrometry | NA | TwinsUK: 53.4 (14.0)  KORA F4: 60.8 (8.8) | TwinsUK: 7  KORA F4: 49 | 24816252 |
| γ-tocopherol | 5,822 | liquid­phase chromatography and gas chromatography separation coupled with tandem mass spectrometry | NA | TwinsUK: 53.4 (14.0)  KORA F4: 60.8 (8.8) | TwinsUK: 7  KORA F4: 49 | 24816252 |

ATBC: Alpha-Tocopherol, Beta-Carotene Cancer Prevention Study; EPIC: European Prospective Investigation into Cancer and Nutrition; InCH: InCHIANTI Study; KORA: The Cooperative Health Research in the Region of Augsburg; NHS: Nurses’ Health Study; PLCO: Prostate, Lung, Colorectal, and Ovarian (PLCO) Cancer Screening Trial; WHAS: Women’s Health and Aging Study.

**^†^**Values are the mean ± SD except for median (interquartile range)

**^††^**Values are the mean ± SD except for median (range)

**Supplementary Table 2** Summary-level genetic association statistics of genetic instruments associated with absolute circulating antioxidants, knee osteoarthritis (OA), hip OA and rheumatoid arthritis (RA)

| Antioxidant | SNP | Effect allele | EAF | Exposure | | |  | Knee OA | | |  | Hip OA | | |  | RA | | |
| --- | --- | --- | --- | --- | --- | --- | --- | --- | --- | --- | --- | --- | --- | --- | --- | --- | --- | --- |
|  |  |  |  | Beta | SE | *P* |  | Beta | SE | *P* |  | Beta | SE | P |  | Beta | SE | *P* |
| Retinol | rs10882272 | C | 0.35 | -0.03 | 0.004 | 6.51E-15 |  | -0.0064 | 0.0095 | 0.50 |  | 0.0288 | 0.01 | 0.02 |  | -0.0198 | 0.02 | 0.4 |
|  | rs1667255 | C | 0.31 | 0.03 | 0.004 | 6.35E-14 |  | 0.0023 | 0.0095 | 0.81 |  | -0.0195 | 0.01 | 0.10 |  | - | - | - |
| β-carotene | rs6564851 | G | 0.36 | 0.149 | 0.015 | 1.60E-24 |  | -0.0068 | 0.0093 | 0.46 |  | 0.0069 | 0.01 | 0.55 |  | 0.0408 | 0.02 | 0.01 |
| Lycopene | rs2232315 | A | 0.03 | 0.74 | 0.15 | 1.26E-06 |  | 0.0169 | 0.0341 | 0.62 |  | -0.0187 | 0.04 | 0.66 |  | 0.0488 | 0.19 | 0.8 |
|  | rs341075 | A | 0.02 | -0.87 | 0.17 | 5.75E-07 |  | 0.0407 | 0.0274 | 0.14 |  | -0.0434 | 0.03 | 0.20 |  | -0.0408 | 0.07 | 0.57 |
|  | rs4635297 | A | 0.08 | 0.26 | 0.05 | 6.46E-07 |  | -0.0084 | 0.0119 | 0.48 |  | 0.0162 | 0.02 | 0.28 |  | 0.0100 | 0.03 | 0.74 |
|  | rs6108801 | C | 0.04 | -0.48 | 0.09 | 4.07E-07 |  | 0.0098 | 0.0249 | 0.69 |  | 0.0351 | 0.03 | 0.26 |  | -0.0953 | 0.06 | 0.11 |
|  | rs7680948 | A | 0.20 | -0.19 | 0.03 | 4.97E-09 |  | -0.0021 | 0.0104 | 0.84 |  | -0.0071 | 0.01 | 0.59 |  | -0.0202 | 0.02 | 0.36 |
| Vitamin C | rs10051765 | C | 0.34 | 0.039 | 0.007 | 3.64E-09 |  | 0.0207 | 0.0098 | 0.04 |  | 0.008 | 0.0124 | 0.52 |  | 0.0408 | 0.0164 | 0.01 |
|  | rs10136000 | A | 0.28 | 0.04 | 0.007 | 1.33E-08 |  | -0.0073 | 0.0103 | 0.48 |  | -0.0183 | 0.013 | 0.16 |  | 0.00995 | 0.0526 | 0.85 |
|  | rs117885456 | A | 0.09 | 0.078 | 0.012 | 1.70E-11 |  | 0.0233 | 0.0164 | 0.16 |  | 0.0296 | 0.0206 | 0.15 |  | - | - | - |
|  | rs13028225 | T | 0.86 | 0.102 | 0.009 | 2.38E-30 |  | -0.0425 | 0.0134 | 0.002 |  | -0.0366 | 0.0169 | 0.03 |  | -0.0408 | 0.0277 | 0.14 |
|  | rs174547 | C | 0.33 | 0.036 | 0.007 | 3.84E-08 |  | -0.0056 | 0.0098 | 0.57 |  | 0.0063 | 0.0122 | 0.61 |  | -0.0583 | 0.0161 | 0.0003 |
|  | rs2559850 | A | 0.60 | 0.058 | 0.006 | 6.30E-20 |  | -0.0112 | 0.0095 | 0.24 |  | -0.0292 | 0.012 | 0.01 |  | 0.00995 | 0.0201 | 0.62 |
|  | rs33972313 | C | 0.97 | 0.36 | 0.018 | 4.61E-90 |  | -0.0048 | 0.0253 | 0.85 |  | 0.0251 | 0.0318 | 0.43 |  | 0.0305 | 0.0452 | 0.5 |
|  | rs56738967 | C | 0.32 | 0.041 | 0.007 | 7.62E-10 |  | -0.005 | 0.01 | 0.62 |  | 0.0154 | 0.0125 | 0.22 |  | - | - | - |
|  | rs6693447 | T | 0.55 | 0.039 | 0.006 | 6.25E-10 |  | -0.0011 | 0.0093 | 0.91 |  | -0.0034 | 0.0117 | 0.77 |  | -0.0101 | 0.0164 | 0.54 |
|  | rs9895661 | T | 0.82 | 0.063 | 0.008 | 1.05E-14 |  | -0.0414 | 0.0123 | 0.001 |  | 0.0189 | 0.0155 | 0.22 |  | 0.00995 | 0.0185 | 0.59 |
| α-tocopherol | rs11057830 | A | 0.15 | 0.03 | 0.01 | 8.2E-09 |  | 0.0076 | 0.0134 | 0.57 |  | -0.0038 | 0.0169 | 0.82 |  | -0.03046 | 0.0394 | 0.44 |
|  | rs2108622 | T | 0.21 | 0.03 | 0.01 | 1.4E-10 |  | -0.0149 | 0.0101 | 0.14 |  | 0.018 | 0.0127 | 0.16 |  | 0.02956 | 0.0246 | 0.23 |
|  | rs964184 | G | 0.15 | 0.04 | 0.01 | 7.8E-12 |  | -0.0001 | 0.0137 | 1.00 |  | -0.0338 | 0.0172 | 0.05 |  | - | - | - |

EAF: effect allele frequency; OA: osteoarthritis; RA: rheumatoid arthritis; SE: standard error; SNP: single nucleotide polymorphism

**Supplementary Table 3** Summary-level genetic association statistics of genetic instruments associated with circulating antioxidant metabolites, knee osteoarthritis (OA), hip OA and rheumatoid arthritis (RA)

| Antioxidant | SNP | Effect allele | EAF | Exposure | | |  | Knee OA | | |  | Hip OA | | |  | RA | | |
| --- | --- | --- | --- | --- | --- | --- | --- | --- | --- | --- | --- | --- | --- | --- | --- | --- | --- | --- |
|  |  |  |  | Beta | SE | *P* |  | Beta | SE | *P* |  | Beta | SE | *P* |  | Beta | SE | *P* |
| Retinol | rs10019071 | A | 0.02 | 0.66 | 0.16 | 3.64E-06 |  | 0.07 | 0.03 | 0.05 |  | 0.09 | 0.04 | 0.04 |  | -0.13 | 0.09 | 0.17 |
|  | rs112293959 | G | 0.03 | -0.43 | 0.13 | 5.70E-06 |  | -0.03 | 0.03 | 0.29 |  | -0.06 | 0.03 | 0.06 |  | - | - | - |
|  | rs113745104 | A | 0.04 | -0.33 | 0.11 | 8.18E-06 |  | -0.03 | 0.02 | 0.27 |  | -0.02 | 0.03 | 0.45 |  | - | - | - |
|  | rs114515641 | G | 0.03 | 0.41 | 0.13 | 7.12E-06 |  | 0.00 | 0.03 | 0.92 |  | 0.04 | 0.03 | 0.24 |  | - | - | - |
|  | rs1153379 | A | 0.93 | -0.32 | 0.08 | 6.10E-06 |  | -0.03 | 0.02 | 0.18 |  | -0.02 | 0.02 | 0.42 |  | - | - | - |
|  | rs117468033 | T | 0.01 | -0.96 | 0.19 | 8.40E-06 |  | 0.02 | 0.05 | 0.67 |  | -0.04 | 0.07 | 0.56 |  | - | - | - |
|  | rs1176744 | C | 0.32 | -0.21 | 0.05 | 3.5E-07 |  | -0.01 | 0.01 | 0.46 |  | -0.01 | 0.01 | 0.47 |  | 0.05 | 0.02 | 0.02 |
|  | rs118025446 | A | 0.03 | -0.48 | 0.12 | 9.84E-06 |  | -0.03 | 0.02 | 0.27 |  | 0.01 | 0.03 | 0.73 |  | - | - | - |
|  | rs12514522 | T | 0.17 | 0.15 | 0.05 | 4.19E-06 |  | - | - | - |  | - | - | - |  | -0.02 | 0.06 | 0.75 |
|  | rs12955464 | G | 0.14 | -0.23 | 0.06 | 3.71E-06 |  | -0.01 | 0.01 | 0.46 |  | 0.01 | 0.01 | 0.69 |  | - | - | - |
|  | rs139726207 | G | 0.04 | 0.37 | 0.11 | 4.46E-06 |  | 0.04 | 0.02 | 0.08 |  | -0.02 | 0.02 | 0.24 |  | - | - | - |
|  | rs149113848 | G | 0.01 | -0.96 | 0.27 | 3.47E-06 |  | 0.03 | 0.05 | 0.56 |  | 0.04 | 0.03 | 0.10 |  | - | - | - |
|  | rs149478645 | G | 0.02 | -0.51 | 0.14 | 1.30E-06 |  | -0.05 | 0.03 | 0.14 |  | 0.05 | 0.07 | 0.46 |  | - | - | - |
|  | rs17005512 | C | 0.17 | -0.22 | 0.06 | 2.77E-06 |  | -0.01 | 0.01 | 0.66 |  | -0.06 | 0.04 | 0.13 |  | - | - | - |
|  | rs1842947 | G | 0.52 | -0.19 | 0.04 | 8.34E-07 |  | 0.01 | 0.01 | 0.24 |  | 0.00 | 0.02 | 0.76 |  | -0.02 | 0.03 | 0.46 |
|  | rs2147337 | G | 0.66 | 0.16 | 0.04 | 9.01E-06 |  | 0.00 | 0.01 | 0.92 |  | 0.00 | 0.01 | 0.92 |  | 0.03 | 0.02 | 0.07 |
|  | rs2367816 | G | 0.77 | 0.23 | 0.05 | 9.46E-06 |  | -0.02 | 0.01 | 0.07 |  | 0.00 | 0.01 | 0.93 |  | -0.04 | 0.02 | 0.08 |
|  | rs2417325 | T | 0.93 | 0.33 | 0.08 | 1.29E-06 |  | 0.03 | 0.02 | 0.09 |  | -0.03 | 0.02 | 0.15 |  | -0.05 | 0.04 | 0.16 |
|  | rs3890033 | C | 0.38 | 0.14 | 0.04 | 8.56E-06 |  | 0.00 | 0.01 | 0.80 |  | 0.00 | 0.01 | 0.79 |  | 0.03 | 0.02 | 0.19 |
|  | rs3898702 | T | 0.20 | -0.22 | 0.05 | 3.02E-06 |  | 0.01 | 0.01 | 0.57 |  | -0.04 | 0.01 | 0.01 |  | -0.01 | 0.02 | 0.64 |
|  | rs4135385 | G | 0.24 | 0.21 | 0.05 | 9.80E-06 |  | -0.02 | 0.01 | 0.03 |  | -0.02 | 0.01 | 0.20 |  | -0.01 | 0.02 | 0.60 |
|  | rs4737112 | G | 0.09 | -0.23 | 0.08 | 9.75E-06 |  | -0.01 | 0.02 | 0.71 |  | -0.02 | 0.02 | 0.33 |  | -0.02 | 0.04 | 0.64 |
|  | rs568632536 | T | 0.03 | 0.53 | 0.14 | 8.08E-06 |  | 0.00 | 0.03 | 0.96 |  | 0.01 | 0.04 | 0.83 |  | - | - | - |
|  | rs58411567 | A | 0.22 | -0.21 | 0.05 | 3.02E-07 |  | 0.00 | 0.01 | 0.89 |  | -0.01 | 0.01 | 0.48 |  | 0.02 | 0.02 | 0.41 |
|  | rs6550239 | A | 0.74 | -0.18 | 0.05 | 4.40E-06 |  | 0.00 | 0.01 | 0.90 |  | 0.03 | 0.01 | 0.04 |  | 0.04 | 0.03 | 0.13 |
|  | rs75308833 | T | 0.02 | -0.49 | 0.15 | 3.51E-06 |  | 0.03 | 0.03 | 0.32 |  | 0.03 | 0.04 | 0.35 |  | - | - | - |
|  | rs7926028 | T | 0.45 | -0.13 | 0.04 | 2.75E-06 |  | -0.01 | 0.01 | 0.17 |  | -0.02 | 0.01 | 0.14 |  | -0.01 | 0.01 | 0.50 |
|  | rs79262371 | C | 0.01 | 0.57 | 0.19 | 8.76E-06 |  | -0.01 | 0.05 | 0.74 |  | -0.02 | 0.06 | 0.76 |  | - | - | - |
|  | rs945817 | A | 0.19 | -0.28 | 0.05 | 6.46E-07 |  | -0.01 | 0.01 | 0.58 |  | 0.01 | 0.01 | 0.39 |  | 0.03 | 0.02 | 0.18 |
|  | rs9546522 | A | 0.38 | 0.13 | 0.05 | 9.13E-06 |  | 0.00 | 0.01 | 0.72 |  | -0.01 | 0.01 | 0.39 |  | -0.03 | 0.02 | 0.15 |
|  | rs9586119 | C | 0.07 | 0.35 | 0.08 | 3.34E-06 |  | 0.01 | 0.02 | 0.60 |  | 0.02 | 0.02 | 0.36 |  | 0.02 | 0.04 | 0.59 |
| Vitamin C | rs11167905 | C | 0.15 | -0.08 | 0.02 | 9.83E-07 |  | -0.02 | 0.01 | 0.07 |  | -0.02 | 0.02 | 0.15 |  | -0.02 | 0.04 | 0.58 |
|  | rs13069990 | T | 0.38 | -0.05 | 0.01 | 4.44E-06 |  | -0.01 | 0.01 | 0.49 |  | 0.00 | 0.01 | 0.84 |  | -0.02 | 0.02 | 0.32 |
|  | rs13103690 | G | 0.46 | 0.05 | 0.01 | 5.20E-06 |  | 0.00 | 0.01 | 0.94 |  | 0.00 | 0.01 | 0.82 |  | 0.05 | 0.02 | 0.01 |
|  | rs2070006 | C | 0.63 | -0.05 | 0.01 | 4.76E-06 |  | 0.01 | 0.01 | 0.51 |  | -0.01 | 0.01 | 0.57 |  | -0.01 | 0.03 | 0.72 |
|  | rs577596 | A | 0.33 | -0.06 | 0.01 | 6.68E-07 |  | 0.01 | 0.01 | 0.27 |  | -0.01 | 0.01 | 0.60 |  | 0.02 | 0.02 | 0.37 |
|  | rs6713914 | C | 0.43 | -0.06 | 0.01 | 3.22E-07 |  | 0.00 | 0.01 | 0.83 |  | -0.02 | 0.01 | 0.20 |  | - | - | - |
|  | rs6826474 | T | 0.04 | -0.14 | 0.03 | 1.56E-06 |  | -0.01 | 0.03 | 0.62 |  | 0.05 | 0.04 | 0.16 |  | 0.11 | 0.06 | 0.07 |
|  | rs6834631 | G | 0.04 | -0.13 | 0.03 | 1.03E-06 |  | 0.00 | 0.02 | 0.89 |  | 0.00 | 0.03 | 0.88 |  | 0.04 | 0.04 | 0.35 |
|  | rs7112460 | T | 0.07 | 0.11 | 0.02 | 1.14E-06 |  | 0.00 | 0.02 | 0.96 |  | 0.00 | 0.02 | 0.89 |  | -0.03 | 0.04 | 0.48 |
|  | rs8057559 | T | 0.03 | 0.14 | 0.03 | 9.10E-06 |  | -0.04 | 0.03 | 0.22 |  | 0.05 | 0.04 | 0.15 |  | 0.02 | 0.08 | 0.80 |
|  | rs808686 | A | 0.61 | 0.06 | 0.01 | 3.01E-06 |  | 0.00 | 0.01 | 0.94 |  | -0.02 | 0.01 | 0.15 |  | -0.01 | 0.01 | 0.45 |
|  | rs8105491 | T | 0.15 | -0.07 | 0.01 | 2.30E-06 |  | 0.00 | 0.01 | 0.76 |  | 0.00 | 0.02 | 0.79 |  | 0.02 | 0.03 | 0.46 |
|  | rs9419004 | C | 0.19 | -0.25 | 0.06 | 6.53E-06 |  | 0.02 | 0.01 | 0.10 |  | 0.00 | 0.01 | 0.78 |  | - | - | - |
|  | rs9606290 | A | 0.24 | 0.16 | 0.04 | 6.32E-06 |  | -0.01 | 0.01 | 0.50 |  | 0.03 | 0.01 | 0.03 |  | -0.03 | 0.04 | 0.41 |
| α-tocopherol | rs10163969 | T | 0.04 | -0.04 | 0.01 | 9.38E-06 |  | -0.01 | 0.02 | 0.78 |  | -0.01 | 0.01 | 0.74 |  | 0.04 | 0.05 | 0.43 |
|  | rs10245705 | T | 0.02 | -0.07 | 0.01 | 1.95E-07 |  | -0.03 | 0.03 | 0.38 |  | -0.11 | 0.01 | 0.01 |  | 0.03 | 0.10 | 0.76 |
|  | rs10935814 | A | 0.10 | -0.04 | 0.01 | 9.44E-06 |  | 0.04 | 0.01 | 0.01 |  | 0.02 | 0.01 | 0.17 |  | 0.01 | 0.06 | 0.87 |
|  | rs11145330 | C | 0.11 | -0.03 | 0.01 | 1.95E-06 |  | 0.02 | 0.01 | 0.17 |  | -0.02 | 0.01 | 0.28 |  | 0.05 | 0.03 | 0.13 |
|  | rs11992435 | G | 0.05 | -0.03 | 0.01 | 6.38E-06 |  | 0.01 | 0.02 | 0.67 |  | 0.02 | 0.01 | 0.49 |  | 0.03 | 0.05 | 0.52 |
|  | rs1404410 | G | 0.21 | 0.02 | 0.01 | 4.57E-06 |  | 0.00 | 0.01 | 0.69 |  | -0.03 | 0.01 | 0.03 |  | - | - | - |
|  | rs1532701 | A | 0.55 | 0.01 | 0.00 | 5.07E-06 |  | 0.01 | 0.01 | 0.47 |  | -0.01 | 0.00 | 0.55 |  | 0.01 | 0.04 | 0.79 |
|  | rs2074731 | A | 0.17 | -0.02 | 0.00 | 2.31E-06 |  | 0.00 | 0.01 | 0.99 |  | -0.02 | 0.00 | 0.20 |  | -0.04 | 0.02 | 0.09 |
|  | rs261342 | C | 0.79 | -0.02 | 0.00 | 5.41E-06 |  | -0.01 | 0.01 | 0.19 |  | 0.00 | 0.00 | 0.80 |  | - | - | - |
|  | rs7238006 | C | 0.07 | -0.03 | 0.01 | 6.77E-07 |  | -0.01 | 0.02 | 0.74 |  | 0.02 | 0.01 | 0.39 |  | -0.03 | 0.04 | 0.46 |
|  | rs7930821 | T | 0.02 | 0.07 | 0.01 | 7.53E-06 |  | -0.01 | 0.03 | 0.82 |  | -0.05 | 0.01 | 0.27 |  | -0.03 | 0.08 | 0.70 |
| γ-tocopherol | rs10077932 | T | 0.14 | -0.04 | 0.01 | 4.08E-06 |  | 0.02 | 0.01 | 0.13 |  | 0.02 | 0.02 | 0.11 |  | 0.02 | 0.04 | 0.62 |
|  | rs1013104 | T | 0.44 | -0.02 | 0.00 | 3.83E-06 |  | 0.01 | 0.01 | 0.34 |  | 0.01 | 0.01 | 0.39 |  | 0.01 | 0.02 | 0.52 |
|  | rs10466757 | T | 0.84 | -0.06 | 0.01 | 9.56E-06 |  | 0.01 | 0.01 | 0.51 |  | -0.01 | 0.01 | 0.58 |  | - | - | - |
|  | rs10492212 | T | 0.16 | -0.03 | 0.01 | 8.66E-06 |  | 0.01 | 0.01 | 0.24 |  | 0.01 | 0.02 | 0.66 |  | -0.03 | 0.03 | 0.25 |
|  | rs10520845 | A | 0.02 | 0.19 | 0.04 | 5.27E-06 |  | -0.03 | 0.04 | 0.48 |  | -0.04 | 0.05 | 0.51 |  | -0.05 | 0.09 | 0.56 |
|  | rs1060467 | G | 0.41 | -0.02 | 0.00 | 2.61E-07 |  | 0.00 | 0.01 | 0.71 |  | -0.01 | 0.01 | 0.25 |  | 0.01 | 0.02 | 0.52 |
|  | rs13336771 | A | 0.17 | 0.06 | 0.01 | 7.39E-06 |  | 0.02 | 0.01 | 0.11 |  | 0.00 | 0.02 | 0.95 |  | -0.02 | 0.02 | 0.35 |
|  | rs261301 | C | 0.87 | -0.03 | 0.01 | 2.06E-06 |  | 0.01 | 0.01 | 0.52 |  | 0.00 | 0.02 | 0.96 |  | 0.01 | 0.04 | 0.78 |
|  | rs2794327 | T | 0.67 | -0.04 | 0.01 | 8.78E-06 |  | 0.01 | 0.01 | 0.45 |  | 0.00 | 0.01 | 0.77 |  | - | - | - |
|  | rs5994305 | G | 0.17 | -0.03 | 0.01 | 7.15E-07 |  | 0.00 | 0.01 | 0.90 |  | -0.02 | 0.02 | 0.26 |  | -0.05 | 0.03 | 0.06 |
|  | rs6821770 | A | 0.14 | 0.04 | 0.01 | 8.92E-06 |  | 0.01 | 0.01 | 0.62 |  | 0.00 | 0.02 | 0.89 |  | -0.03 | 0.03 | 0.29 |
|  | rs7038957 | C | 0.17 | 0.03 | 0.01 | 3.86E-06 |  | 0.00 | 0.01 | 0.73 |  | 0.03 | 0.02 | 0.06 |  | -0.03 | 0.02 | 0.21 |
|  | rs7350776 | G | 0.30 | -0.02 | 0.01 | 3.86E-06 |  | 0.00 | 0.01 | 0.66 |  | 0.00 | 0.01 | 0.93 |  | - | - | - |

EAF: effect allele frequency; OA: osteoarthritis; RA: rheumatoid arthritis; SE: standard error; SNP: single nucleotide polymorphism

**Supplementary Table 4** Mendelian randomization (MR)-pleiotropy residual sum and outlier (MR-PRESSO) analysis for association of genetically determined per unit increase in absolute circulating levels of vitamin C with risk of knee osteoarthritis (OA) and rheumatoid arthritis (RA)

| Exposure | Outcome | No. of outliers | Raw estimate | *P*-value | Outlier-corrected estimate | *P*-value |
| --- | --- | --- | --- | --- | --- | --- |
| vitamin C | Knee OA | 1 | 0.90 (0.77-1.06) | 0.25 | 0.99 (0.88-1.12) | 0.40 |
| vitamin C | RA | 1 | 0.98 (0.71-1.34) | 0.89 | 1.05 (0.83-1.33) | 0.70 |

OA: osteoarthritis; RA: rheumatoid arthritis
